# Older Women’s Loneliness and Depression Decreased by a Reminiscence Program in Times of COVID-19

**DOI:** 10.3389/fpsyg.2022.802925

**Published:** 2022-02-21

**Authors:** Sacramento Pinazo-Hernandis, Alicia Sales, Dolores Martinez

**Affiliations:** Faculty of Psychology, University of Valencia, Valencia, Spain

**Keywords:** loneliness, reminiscence, nursing home, older women, COVID-19

## Abstract

The confinement caused by the current COVID-19 pandemic protects physical health, but in turn, has a long-lasting and far-reaching negative psychosocial impact; anxiety, stress, fear and depressive symptoms. All of these have a particular impact on vulnerable older people, putting them at serious risk of loneliness. Women report feeling lonelier than men, affecting women to a greater extent. The present study aims to analyze the efficacy of an integrative reminiscence intervention in older women living in nursing homes to reduce the effects of loneliness and depression after COVID-19. 34 older women living in nursing homes are included into study and were divided into intervention group (*N* = 14) and control group (*N* = 20). Results showed a significant reduction in perception of loneliness, depression and better positive affects, after the intervention. The pandemic has not yet finished and the most affected group has been the people living in nursing homes. These results show the need for evidence of interventions that can help the recovery of these people who have been so affected. The effects of loneliness during confinement and its psychological effects can be mitigated through such programs.

## Introduction

The pandemic has affected both directly and indirectly to older adults in nursing homes, through COVID-19 or social isolation (Miller, 2020). There is a great deal of research supporting the fact that the isolation, social distancing, social disconnection, and loneliness are related to depression and anxiety. Self-perceived social disconnection and perceived isolation predict greater symptoms of depression and anxiety (Mukhtar, 2020). Social isolation is one of the major contributors to mortality in older adults (Holt-Lunstad et al., 2015) and has a measurable impact on health outcomes (Luo et al., 2012; Friedler et al., 2015).

This isolation during the pandemic, coupled with the absence of relationships, social and therapeutic activities, has generated a significant worsening of the cognitive, functional and emotional older adults’ capacities (Pinazo-Clapés et al., 2020; Pereiro et al., 2021). Taylor S. et al. (2020) state that all the measures of physical distancing have a direct impact and a long-term impact on physical, emotional and older adults’ social aspects.

Social distancing and dependence on other people for the performance of daily living activities make older adults even more vulnerable (Han and Mosqueda, 2020). The awareness of vulnerability itself generates anxiety and psychiatric symptoms (Naarding et al., 2020), and many of the older adults living in nursing homes are in this state of vulnerability. Campbell-Enns et al. (2020) state that the reduction of social interaction and reduced quality of contacts in nursing homes involves the suspension of visits, communal meals in dining rooms and group activities in common rooms. The isolation of older adults have experienced since March 2020 has severely impacted them requiring urgent actions to limit the mental health consequences, especially anxiety and depression (Armitage and Nellums, 2020). Some studies conclude that being an older woman, compared to being an older man (Cole and Dendukuri, 2003) as well as lack or loss of close social contacts (Djernes, 2006) are risk factors for mental health. According to Plagg et al. (2020) the fear, stress, loneliness and social isolation of older adults during the pandemic may undermine resilience and, as a consequence, further compromise subjective and psychological well-being.

COVID-19 pandemic and different measures indicated by Health Services (physical distancing, lockdown, and stay-at-home) are expected to intensify feelings of loneliness. Earlier analyses conducted in previous pandemics have demonstrated increases in loneliness, anxiety and depression from quarantine-induced social isolation (Hawryluck et al., 2004; Brooks et al., 2020; Rana et al., 2020).

Older adults are at higher risk of anxiety and depression when put in situations of social disconnection (Armitage and Nellums, 2020), and different studies showed a significant increase of anxiety in society during this pandemic (Huang and Zhao, 2020; Qiu et al., 2020; Teufel et al., 2020).

Nursing homes are mainly inhabited by very old people (average age 83 years). Most people who decide to live in a nursing home do so because they need continuous, specific and long-term care. Many of them have cognitive disease. Most of them are women in a situation of vulnerability and dependence (Butcher et al., 2018). Many studies have found gender differences in loneliness, finding higher levels of loneliness and depression in women although all studies do not agree with this and some even find greater loneliness in men (higher levels of loneliness in males, Cooney and Dunne, 2001) or higher levels of loneliness in females (Pinquart and Sörensen, 2001); also similar levels in women and men (Maes et al., 2019).

Other factors of social exclusion co-occur in older women (greater dependency, living alone for more years, lower economic level). Depression is a serious problem in older women living in nursing homes, but it is not only a mental health problem; it can also affect physical and functional health (Cahoon, 2012). Depressive symptoms are most prevalent in older women in long-term care facilities and they are more vulnerable to stressful events (Lampert and Pereira, 2015). The presence of depression is often found to be related to factors concerning the environment in which the older women reside.

Reminiscence interventions, that uses a recall of past events, moments and important people in one’s life, have been used to review the life experiences of older people, promote positive feelings by sharing life experiences, and give meaning to lives lived by integrating present and past (Westerhof and Bohlmeijer, 2014). Group reminiscence interventions, can reduce depression and anxiety levels, and improve psychological wellbeing in older adults living in nursing homes with mild stage Alzheimer’s (İnel and Simsek, 2019) and moderate stages (Hsieh et al., 2010; Duru and Kapucu, 2016; Lök et al., 2019).

Tam et al. (2021) conducted a systematic review and meta-analysis on the effectiveness of reminiscence interventions to improve psychological well-being in older adults aged 60 years or older without cognitive impairment; concluding that reminiscence intervention significantly reduces depressive symptomatology and improves life satisfaction, with positive effects on self-esteem, psychological well-being and happiness. Reminiscence therapy, has shown positive health outcomes for older adults living in community and nursing homes (Faith, 2018; O’Philbin et al., 2018): Reduced depressive symptoms (Watt and Cappeliez, 2000; Karimi et al., 2010; Melendez et al., 2015; Soniya, 2015; Lopes et al., 2016; Henkel et al., 2017; Satorres et al., 2018; Siverova and Buzgova, 2018), reduced anxiety (Haslam et al., 2014; Lopes et al., 2016), improve wellbeing and life satisfaction (Gallagher and Carey, 2012; Haslam et al., 2014; Smiraglia, 2015; Viguer et al., 2017), improve quality of life (Westerhof et al., 2004), social engagement (Bohlmeijer et al., 2008; Siverova and Buzgova, 2018).

Research by Melendez et al. (2015), found the effectiveness of integrative reminiscence therapy as a useful intervention in people with dementia, finding it to be especially effective in reducing depressive symptoms and improving psychological well-being.

Non-pharmacological therapies, such as reminiscence intervention, can help reinterpret lives and bring meaning to them (Shropshire, 2020).

The consequences of the COVID-19 pandemic on psychological distress often go unnoticed (Zhang et al., 2020). In relation to this fact, Rodríguez-Rey et al. (2020) conducted a study on the psychological impact of COVID-19 in the Spanish population, concluding that more than 36% of the participants showed significant psychological distress due to the current health crisis. However, few studies have focused on interventions to alleviate the psychological effects of COVID-19 in older adults living in nursing homes carried out during times of pandemic, immediately after the confinement. Regardless of whether isolation and quarantine induce posttraumatic stress disorder, public health officials must be cognizant of and prepared to supply appropriate emotional and social support to persons subject to isolation or quarantine (Savage et al., 2021).

The main objective of this study was to investigate how the dimensions selected (loneliness, depression, anxiety, affect) change over time after an intervention and follow-up based on a reminiscence therapy program in a sample of older women who lived in a nursing home in order to show its efficacy in alleviating the effects of variables directly related to and affected by confinement and COVID-19 pandemic. We hypothesize that the intervention will produce a significant effect on the intervention group improved anxiety and depression levels, less negative affectivity and higher levels of positive affectivity.

## Materials and Methods

### Participants

Participants included 34 older women living in nursing homes in Spain. The sample is not very large but the profile is similar to the one that could be found in any nursing home. The centers of a corporation (five) were randomized to determine where the intervention program would be administered. Reminiscence therapy (RT) was carried out in one of the centers. This program was offered to older adults and only women volunteered to participate. Women are the majority in nursing homes, and they are also the most involved in group programs in the centers.

All participants met the inclusion criteria and gave informed consent to take part in the study.

The inclusion criteria were: Older women living in the nursing home at the time of the study, having been there during the confinement state; maintaining preserved cognitive functions or presenting cognitive impairment (scores above 23 on the Mini Mental Scale Examination, MMSE; Folstein et al., 1975). The MMSE Spanish version gives a brief, standardized analysis of cognitive state, estimating the possible existence and severity of cognitive impairment. Screening instrument consists of 30 items grouped into five cognitive domains: orientation, registration, attention-calculation, recall, and language including the item of visual construction. The MMSE score range is from 0 to 30, and his cutoff point for mild cognitive impairment is 23 points.

The exclusion criteria were advanced diseases with terminal criteria (renal, cardiac, respiratory failure, terminal or active neoplasms, etc.), temporary stays (that did not allow follow-up), presence of sensory deficits (that prevented participation, evaluation and follow-up), and severe psychiatric disorder in the medical history.

Before starting the reminiscence program and always by the same trainer, participants were evaluated (MMSE and Depressive and Anxiety Scale) individually before being assigned to groups: Intervention Group (IG) and Control Group (CG). After this first evaluation of the 43 people tested, 8 were excluded based on inclusion/exclusion criteria, 3 for presenting MMSE scores lower than below the cutoff point established by scale as mild cognitive impairment, and 5 for presenting depressive symptoms (Goldberg Scale). The participants (*N* = 35) were randomly assigned to either the intervention group (IG; *N* = 15) and control group (CG; *N* = 20). Members of the intervention group had to attend at least 90% of the sessions. For health reasons, one participant did not have 90% attendance and were excluded from the analyses, leaving a final sample size of 34 participants (*N* = 14 in the intervention group; *N* = 20 in the control group).

To evaluate the program’s effects, the experimental design included pretest, posttest, and a follow-up evaluation after 3 months, in an IG and a CG. Members of the CG were on a waiting list to participate in the RT at the end of the study.

The average age was 88, 36 years (*SD* = 3.77) in the IG and 87.20 years (*SD* = 8.05) in the control group. In terms of civil status, 92.2% of the IG were widowed and 7.1% single; in the CG, those percentages were 75% widowed, 15% single, 10% married. Related to education level, 28.6% of the IG had not completed elementary school, 21.4% had completed elementary school, 28, 6% middle or secondary school and 21, 4% had completed university level; in the CG, those percentages were 10, 20, 55, and 15%, respectively.

Tests for homogeneity revealed no significant differences between groups at pre-intervention : age [*t*(32) = 0.499, *p* = 0.621], marital status (U Mann-Whitney *z* = 138.5, *p* = 0.937), and educational level (U Mann-Whitney *z* = 117.5, *p* = 0.405), showing no significant differences.

### Instruments

In addition to collecting sociodemographic data, some tests and scales were administered to take pre- and post-intervention measures:

-Loneliness was measured using the de Jong Gierveld Loneliness Scale (dJGLS) (De Jong Gierveld and Kamphuis, 1985), based on the cognitive model of loneliness which states that the greater the discrepancy between desired and actual social relationships, the greater the experience of loneliness. It is based on the three-dimensional conceptualization of loneliness: type of lack, the nature and intensity of the relationships that the subject lacks; time perspective, temporarily experienced loneliness vs. permanent loneliness and emotional characteristics, lack of positive feelings vs. the presence of negative feelings (Pinazo-Hernandis and Bellegarde, 2018). It is composed of 11 items and the loneliness score ranges from 0 (No loneliness) to 11 (Extreme loneliness). dJGLS has demonstrated a good reliability (Cronbach’s α = 0.86).

-To tap depressive and anxiety symptomatology, the Goldberg Depressive and Anxiety Scale (GADS) (Goldberg, 1979), was utilized. It is a hetero-administered questionnaire with two subscales of 9 binary (yes/no) items: anxiety scale composed of the first 9 items and depression scale composed by the last 9 items. The initial questions of each subscale are conditioning questions. At least two affirmative answers are required for questions 1–4 to discontinue the anxiety subscale and one positive answer is needed from questions 10–13 to continue answering the depression subscale.

The cut-off points for the anxiety subscale are greater than or equal to 4 points and 2 or more points for the depression subscale, being 9 points as the highest possible value for each subscale. Higher point values indicate a more severe problem. Internal consistency reliability was calculated for anxiety scale (Cronbach’s α = 0.62) and depression scale (Cronbach’s α = 0.53).

-The Positive and Negative Affectivity Scale (PANAS) (Watson et al., 1988) was used to measure positive affectivity or emotionality and negative affectivity or emotionality. It consists of 10 positive (e.g., interested) and 10 negative items (e.g., distressed). Respondents are asked to rate the extent to which they have experienced each particular affect within a specified time period on a scale from 1 = very slightly to 5 = extremely as to how felt in general. The scores on the subscales (positive or negative affect) range from 10 to 50, with the lowest score being 20 and the highest 100. High scores on each subscale indicate high positive or negative affect. It is one of the most widely used to study affect and its relationship with different areas of people’s functioning, particularly in relation to health (Taylor S. et al., 2020), with positive affect being a protective element and negative affect a risk factor for illnesses (Krijthe et al., 2011; Taylor S. S. et al., 2020). The obtained internal consistency reliability for this measure was calculated as a = 0.88 for positive affect scale and = 0.76 for negative affect scales in the present study.

### Procedure

RT was carried out in a group setting, which is the most recommended by different researchers (Watt and Cappeliez, 2000; Westerhof et al., 2004; Karimi et al., 2010; Haslam et al., 2014; Smiraglia, 2015; Soniya, 2015; Lopes et al., 2016; Hanaoka et al., 2018; Satorres et al., 2018; Siverova and Buzgova, 2018). The intervention consists of a reminiscence program, directed by a psychologist. The RT was conducted in a suitable room where it was possible to maintain the physical distance of 2 meters (6 feet). The sessions were always held in the same room, on the same day of the week and at the same time of the day. During July 2020 the pretest measure was taken and the post-test measure in October 2020. Finally, the follow-up measure was taken in January 2021 coinciding with the fourth wave of the COVID-19 pandemic with a lot of people affected by SARS-COV-2 in the center, lockdown in their rooms and isolating during most of the day.

Integrative reminiscence is a process to reconcile the discrepancy between the ideal and the real, to know and identify the lines that give continuity to the lives of people in the past and present, finding the explanations, meanings, sense that people give to their lives as a whole (Watt and Cappeliez, 2000).

RT consisted of 10 sessions, carried out in 6 weeks (2 sessions per week), lasting 90 min. The procedure was standardized with self-made guidelines used to address the topics planned for the session. RT was conducted by strategies, dynamics and activities through several eliciting stimuli. In each session a different theme is worked on, favoring the memory of different emotional valences: (1) Optimal experience, (2) lowest moment, (3) decisive moment (turning point), (4) earliest memory of childhood, (5) memory of adolescence, (6) memory of adulthood, (7) significant people, (8) future script and meaning of life, (9) stressors, problems and solutions, (10) beliefs and values. RT was developed by a trained facilitator (psychologist, DMC) and supervised by two researchers (SPH and ASG). The participants in IG were 14 women.

### Data Analysis

We performed *t*-test analysis, a mixed two ANOVA analysis factors with repeated analysis on the last one (time), and analysis of variance. All analyses were carried out using the SPSS 26 statistical package.

We performed *t*-tests for independent samples and chi-squared tests to determine whether or not the groups were homogenous prior to treatment. To analyze the intervention’s effects, repeated measures analysis of variance was conducted, applying the Bonferroni correction (α < 0.05). Simple effects as well as interaction effects (group × time) were examined. The level of statistical significance employed was *p* < 0.05.

## Results

Firstly, simple effects analysis of the perception of loneliness revealed no significant differences between the groups in the pre-measurement scores, but there were significant differences in the post and follow-up measurements (see Table 1). Furthermore, the time-group interaction revealed significant differences [*F*(2, 54) = 12.45; *p* = 0.000; η^2^ = 0.316].

**TABLE 1:** Loneliness, anxiety and depression and affect means at the three time points and univariate statistics for comparison between groups.

Time	IG	CG	*F*	*p*	η^2^
Loneliness pre-intervention	3.25	4.52	1.13	0.258	0.047
Loneliness post-intervention	1.33	6.70	30.42	0.000	0.530
Loneliness follow-up	5.83	10.29	53.08	0.000	0.663
Anxiety pre-intervention	4.00	2.41	4.35	0.047	0.139
Anxiety post-intervention	2.91	3.82	2.07	0.162	0.071
Anxiety follow-up	3.08	4.88	2.77	0.108	0.093
Depression pre-intervention	2.83	2.17	0.974	0.332	0.035
Depression post-intervention	1.91	3.05	3.10	0.089	0.103
Depression follow-up	3.00	4.58	6.92	0.018	0.189
Negative affect pre-intervention	19.41	18.23	0.371	0.547	0.014
Negative affect post-intervention	16.58	23.70	8.26	0.008	0.234
Negative affect follow-up	21.83	30.94	22.04	0.001	0.450
Positive affect pre-intervention	39.66	39.52	0.003	0.957	0.000
Positive affect post-intervention	39.50	30.94	12.35	0.002	0.314
Positive affect follow-up	35.15	26.53	14.73	0.001	0.353

In addition, when analyzing the evolution of each group independently over time, significant differences in loneliness were obtained in both the intervention group [*F*(2, 26) = 25.62; *p* = 0.002; η^2^ = 0.663] and the control group [*F*(2, 26) = 38.95; *p* = 0.000; η^2^ = 0.750] over the three time periods. In the intervention group, a significant decrease in score was observed between the pre and post measurement (T1 = 3.25; T2 = 1.33; *p* < 0.001), and a significant increase between the post and follow-up measurement (T3 = 5.83; *p* = 0.000); in the control group, a significant increase was observed both in the mean pre and post (T1 = 4.52; T2 = 6.70; *p* = 0.000), as well as between the post and follow-up measurement (T3 = 10.29; *p* = 0.000).

Secondly, and in order to analyze the effect of the RT on Depression and Anxiety on the Goldberg scale, simple effects showed a significant difference between the groups in the pre but not in the post and follow-up measures in anxiety, while in depression no significant difference was observed in the pre and post measures but in the follow-up measure (see Table 1). A significant effect of the intervention was observed for the time-group interaction on anxiety [*F*(2, 54) = 22.22; *p* = 0.000; η^2^ = 0.452] and depression [*F*(2, 54) = 12.61; *p* = 0.000; η^2^ = 0.318].

When analyzing each group over time, significant differences in anxiety were obtained in the intervention group [*F*(2, 26) = 16.86; *p* = 0.000; η^2^ = 0.565] and control [*F*(2, 26) = 35.22; *p* = 0.000; η^2^ = 0.730] over the three times. Specifically, and as for intervention group is a significant decrease in scores from pre-intervention mean to post-intervention mean (T1 = 4.00; T2 = 2.91; *p* = 0.025) and a significant increase from post to follow-up mean (T3 = 3.83 = *p* = 0.000). As for the control group, there was a significant increase from pre-intervention to post-intervention mean (T1 = 2.41; T2 = 3.82; *p* = 0.000) and from post to follow-up (T3 = 4.88; *p* = 0.000).

On the other hand, significant differences were obtained in depression the intervention group [*F*(2, 26 = 17.67; *p* = 0.000; η^2^ = 0.576] and control [*F*(2, 26) = 41.25; *p* = 0.000; η^2^ = 0.760] throughout the three times in depression. In the treatment group there is a slight marginally significant decrease (T1 = 2.83; T2 = 1.91; *p* = 0.076) between the pre and post measure but then there is a significant increase in the follow-up measure (T3 = 3.00; *p* = 0.000). In the control group there is a significant increase from the pre to post mean (T1 = 2.17; T2 = 3.05; *p* = 0.035) and from the post to the follow-up measure (T3 = 4.58; *p* = 0.000).

Third and finally, the effect on affect was analyzed with the measure Positive and Negative Affect general (more generic and representative of their global affective states). When analyzing the simple effects of negative affect, it was observed that there were no significant differences between groups in the pre-measurement scores, but there were significant differences in the post measurement scores and in the follow-up measurement scores (see Table 1). The time-group interaction revealed a significant effect [*F*(2, 54) = 12.93; *p* = 0.000; η^2^ = 0.324].

In addition, when analyzing the evolution of each group independently over time, significant differences were obtained both in the intervention group [*F*(2, 26) = 8.00; *p* = 0.002; η^2^ = 0.381] and in the control group [*F*(2, 26) = 41.81; *p* = 0.000; η^2^ = 0.763] throughout the three times, finding a maintenance of the score in the intervention group between the pre and post mean (T1 = 19.41; T2 = 16.58; *p* = n.s) and a significant increase in the score between the post and follow-up measurement in the intervention group (T3 = 21.83; *p* = 0.001). in the control group a significant increase was observed from the pre to the post measurement (T1 = 18.23; T2 = 23.70; *p* = 0.004) and between the post and follow-up measurement (T3 = 30.94; *p* = 0.000).

Regarding positive affect, first the study of simple effects confirmed that there were no differences between the groups in the scores in the pre time but there were differences in the post and follow-up (see Table 1). Secondly, the time-group interaction showed a significant effect [*F*(2, 54) = 15.80; *p* = 0.000; η^2^ = 0.369].

In addition, and when analyzing the evolution of each group independently over time, significant differences were obtained in both the intervention group [*F*(2, 26) = 15.45; *p* = 0.000; η^2^ = 0.543] and the control group [*F*(2, 26) = 43.35; *p* = 0.000; η^2^ = 0.769] throughout the three times, finding a maintenance of the score in the intervention group between the pre and post mean (T1 = 39.66; T2 = 39.50; *p* = n.s) and a significant decrease between the post mean and the follow-up (T3 = 35.15; *p* = 0.000), while in the control group a significant decrease was observed from the pre to the post measure (T1 = 39.52; T2 = 30.94; *p* = 0.000) and between the post measure and the follow-up (T3 = 26.53; *p* = 0.000).

## Discussion

The COVID-19 pandemic has had a serious impact on society in general, but very hard on older adults, especially those living in nursing homes. Isolation, physical distancing between people, prohibition of family visits, routine walks and outings, have had a direct impact on physical, emotional and social aspects that are very relevant for the health of the older adults (Taylor S. et al., 2020), with repercussions in the increase of feelings of loneliness, fear or helplessness and in the rates of depression and anxiety (Taylor S. et al., 2020). The situation experienced during the pandemic caused emotional distress and anxiety. The impact of loneliness has been exacerbated by the COVID-19 pandemic in all parts of the world (Rodney et al., 2021). The effects of the COVID-19 pandemic have been many, but at the societal level, many older adults who are socially isolated and live in community settings have been a high-risk group. People living in nursing homes in different countries around the world have had to cope with restrictions to social interaction: cancelation of family visits and face-to-face contact, social distance,. Lack of social support is detrimental to the physical and psychological well-being of nursing home residents and can lead to an increased risk of morbidity and mortality.

We do not yet know the long-term effects of the COVID-19 quarantine on the health of older adults, although if we look at what previous studies say about the consequences for mental health in previous quarantines, they have found increased depression, emotional disturbances, low mood, sleep problems (Courtin and Knapp, 2017).

Although there is not a lot of information about what happened, some studies show that the prevalence of anxiety and depression during COVID-19 outbreak, varies across the studies, having a wide range for anxiety from 8.3 to 49.7% and for depression from 14.6 to 47.2%, in research made by Lei et al. (2020) and Losada-Baltar et al. (2021), respectively.

The aim of the study was to examine the efficacy of reminiscence in older women during the COVID-19 pandemic. Different research shows that reminiscence therapy has effects on depressive symptoms, improves psychological well-being (dimensions of self-acceptance, autonomy, mastery of the environment and positive relationships with others), promote greater life satisfaction (Bohlmeijer et al., 2008; Gonzalez et al., 2015; Westerhof and Slatman, 2019), increases adaptation to stress that are happening as a normal aging process throughout life (Satorres et al., 2021) and even a decrease in behavioral and psychological symptoms of dementia (Park et al., 2019). In addition, RT has been used to facilitate adjustment to aging (Satorres et al., 2018) and improve autobiographical memory (Melendez et al., 2017).

The findings indicated significant improvement in perception of loneliness, depression and affectivity.

Firstly and regarding loneliness, longitudinal studies can provide solid evidence of temporal changes. There are not many longitudinal studies in times of pandemic. Luchetti et al. (2020) found that older adults showed a slight increase in loneliness in late March 2020 after social distancing measures implemented in the United States compared with the baseline assessment in January/February.

In a survey of older adults, Li and Wang (2020) found high levels of loneliness during the COVID-19 pandemic: lonely some of the time (34.8% of respondents) and feeling lonely always or often (8.3%). Women reported significantly higher levels of loneliness, despite controlling for factors like living alone, health status and caregiving. Our findings are similar to the published literature to date: Li and Wang (2020) found 22.4% of older adults reported feeling lonely sometimes and 4.1% of older adults reported feeling lonely often, in the first 4 weeks of lockdown.

Loneliness and health are related. Social isolation and loneliness are important risk factors and can be a cause but also a consequence of poor physical and mental health (Wu and McGoogan, 2020). Already prior to the COVID-19 pandemic, loneliness and social isolation were recognized as public health problems related to an increased risk of morbidity and mortality.

Social isolation and loneliness has been associated with a higher prevalence of vascular and neurological diseases (Holt-Lunstad et al., 2015).

On the other hand, there is evidence of the relationship between social isolation and loneliness with increased inactivity, increased disease and increased risk of mortality (Holt-Lunstad et al., 2010; Schrempft et al., 2019). The quarantine and subsequent months in this pandemic time have produced in people of all age groups, changes in lifestyle; these changes have affected older people even more, by reducing social interactions, possibilities of participation, etc., and all this has negatively affected physical and mental health (Vahia et al., 2020).

The decreasing of social interaction produced by social distancing could have a negative impact on mental and physical health in older adults because it has limited the social participation. Social participation increase interaction with other people producing effects in sense of belonging, self-esteem, affectivity, and positive emotions.

In relation with present study, participants’ perception of loneliness, after the intervention, presented less experience of loneliness. Relationships with others provide throughout life a sense of belonging, inclusion, connectedness, provide support, which is an excellent buffer against stressful life events and anxiety (Flett et al., 2019), as has been the case during the pandemic (Flett and Zangeneh, 2020). There is a significant relationship between loneliness and isolation (Berg-Weger and Morley, 2020; Plagg et al., 2020), so through this therapy such feelings of loneliness could be alleviated.

Secondly and in relation to depression, and following the line of other previous studies (Park et al., 2019), our results show how reminiscence therapy during the pandemic improved depression levels. Moreover, after the intervention, the participants in our study presented less negative affectivity and higher levels of positive affectivity. Following the trifactorial model of reminiscence functions (self-positive, self-negative and prosocial functions) proposed by Cappeliez and O’Rourke (2006), and confirmed and validated in Spanish sample by Ros et al. (2016), these same authors show that the self-positive factor is negatively related and the self-negative factor is positively related to mental health.

Van Bogaert et al. (2016) results showed that in comparison with a control group who received usual care, residents who received the reminiscence therapy showed significant less depressive symptoms. Their program focus on family, home, community or life role aiming to impact participants’ cognition, well-being and behavior as well as to increase facilitators’ supportive role and experiences as a change agent in the reminiscence process.

Thirdly and in relation to anxiety, own results cannot conclude an effect of the intervention on this variable. When analyzing simple effects show a significant difference between the groups in the pre but not in the post and follow-up measures in anxiety. The IG showed a significantly higher mean pre-intervention anxiety than the CG. This indicates that there is no homogeneity in anxiety in the pre-intervention measure. These results cannot indicate that the significant decrease between pre and post anxiety measurements in the IG is caused by the intervention.

The effects of quarantine (physical distancing, absence of relationships, social and therapeutic activities, etc.) have a direct impact on physical, emotional and older adults’ social aspects (Taylor S. et al., 2020). Oder adults’ isolation has severely impacted them with mental health consequences, especially anxiety and depression (Armitage and Nellums, 2020). For this reason, urgent actions as psychological interventions were necessary during pandemic to reduce negative psychological consequences. Since RT has been shown to have positive effects on these psychological aspects, this study proposed to RT during pandemic to analyze these effects.

Our research has some limitations. There is a small sample, but we have analyzed the effects of RT using time series data. Other limitation is that we estimated prevalent cases at the moment of our survey was made but there would have been interesant have had previous data that could assess how existing cases of anxiety or depression changed along pandemic times. In the present study a follow-up was carried out only 3 months after the intervention. But the follow-up measurement coincided with another wave of the COVID-19 pandemic (fourth wave), so it has not been possible to show results of the maintenance of the improvement. It shows the impact that the higher incidence rate can cause and with it the increase of restrictions and isolation. For this reason, a longer follow-up period (6 or 12 months) could have been used to see if the improvement is maintained in the long term.

## Conclusion

This study showed the effect of the RT developed during pandemic period.

The study identified the capacity of an intervention based on an integrative reminiscence program applied to randomly selected older women of two nursing homes, randomized in two study groups—intervention and control. In this study one trained facilitator performed 10 group sessions.

COVID-19 has changed the interest of society in general in nursing homes and older adults’ care. A debate has been opened that no longer concerns only professionals, experts or family members, but society in general. Person-centered care is the highest quality model of care for the elderly, especially nursing home care. At this model, different aspects are important as: knowing the person; or providing meaningful activities. A program like this allows staff nurses and caregivers to learn about life and preferences of older adults, improve communication and made individualized interventions.

Integrative reminiscence intervention has made a significant effect on loneliness, decreasing in scores over the three time measures. Integrative reminiscence intervention has reduced depressive levels on the intervention group. Significant differences were observed in positive affect. These three variables have been shown to be affected during the pandemic in people living in nursing homes, and are important variables that determine well-being and mental health.

The most appropriate psychological and physical recommendations are not known due to lack of evidence.

At this time of recovery after so long of pandemic, where the most affected group has been the people living in nursing homes, it is urgent to carry out intervention programs that have shown their effectiveness to help as soon as possible the recovery of these people who have had such a hard time for so many months.

The loneliness experienced for so long from social distance has produced effects on the psychological, cognitive and even physical health of people living in nursing homes. This program is one of the few that we have been able to carry out during this year inside the nursing homes. We do not know if any other intervention has been carried out, but we have not found scientific articles that speak of programs and results during this period of pandemic.

Future studies should be conducted with longitudinal designs, with larger numbers of people and taking into account more emotional, mental, social and physical health variables.

Our research shows the change associated to pandemic but these data must continue be updated because the pandemic has not yet finished.

## Data Availability Statement

The original contributions presented in the study are included in the article/supplementary material, further inquiries can be directed to the corresponding author.

## Ethics Statement

The studies involving human participants were reviewed and approved by the University of Valencia. The participants provided their written informed consent to participate in this study.

## Author Contributions

SP-H: conceptualization and developed the theoretical idea, literature revision, methodology, reviewing, and supervision. SP-H and AS: data curation, writing-original draft preparation, and editing. DM: writing-original draft preparation and reviewing. All authors contributed to the article and approved the submitted version.

## Conflict of Interest

The authors declare that the research was conducted in the absence of any commercial or financial relationships that could be construed as a potential conflict of interest.

## Publisher’s Note

All claims expressed in this article are solely those of the authors and do not necessarily represent those of their affiliated organizations, or those of the publisher, the editors and the reviewers. Any product that may be evaluated in this article, or claim that may be made by its manufacturer, is not guaranteed or endorsed by the publisher.
